# Deportment of Blood-Clot and Blood-Deposits in Vessels and Tissues

**Published:** 1874-10

**Authors:** Frank A. Ramsey

**Affiliations:** Knoxville, Tenn.


ATLANTA

MEDICAL AND SURGICAL JOURNAL.

Vol. XII.] OCTOBER—1874. [No. 7.

### Original Communications.

### DEPORTMENT OF BLOOD-CLOT AND BLOOD-DEPOSITS IN VESSELS AND TISSUES.

By FRANK A. RAMSEY, M.D.

*Read before the Knox County Section of the East Tennessee Medical Society, August* 21, 1874.

At the fifty-sixth regular session of the Gynæcological Society of Boston occurred "a passage" between two members, which is thus recorded:

"Dr. Dutton had always supposed that the tendency of hæmatocele was to absorption rather than to suppuration.

"Dr. Warren did not agree with Dr. Dutton's views of the subject, and believed he was supported by nine-tenths of the members of the profession.

"Dr. Dutton reiterated his views, and declared himself determined to stand by them, Dr. Warren's opinions and those of nine-tenths of the profession notwithstanding. Dr. Dutton further stated that he thought it generally understood that suppuration was the distinctive sign between hæmatocele and pelvic abscess."

Dr. Dutton manifested the spirit of "a plucky little fellow" in his declaration to "withstand" nine-tenths of the profession, admitting the truth of Dr. Warren's affirmation regarding them; but would it not have been more in accord with the spirit of earnest inquiry if Dr. Dutton had challenged the affirmation, and called for specific citation of reliable observers and students holding the opinion in common with Dr. Warren on the point involved? Such a course would have condensed information and redounded to the benefit and satisfaction of very many who are now more at loss than before discussion occurred and the records were published. The transactions of the Gynæcological Society of Boston are read, repeatedly read, by members of the profession throughout the country, for the purpose of getting definite knowledge; and a mere conflict of opinion between two of its members serves only to make "doubt deeper and make spots black that were but cloudy." Hence I feel inclined to prosecute these disputants of the fifty-sixth session of the Gynæcological Society for ethical direlection. The one who, in his extremity and anxiety, was in much haste to fortify an averment, for, without tremor or consideration, but boldly placing nine-tenths of the profession, without their permission, or without specific citation of their recorded opinion, on the doctrinal platform he had erected. It is as common as it is easy for medical men, when casually conversing on a mooted point belonging to their daily avocations, to dogmatically asseverate each his own enunciation to be the opinion held by the majority of medical practitioners, if, indeed, not *savants*. But such dogmatism, as is positivism always, by men who stand in awe of the immensity of learning to be attained, and admit their own insufficiency to read the profound mysteries almost hourly presented for them to consider, is occasion for regret. It is not usual for the profounder students of healthy and diseased, natural and morbid, actions and results in the human economy to be unqualified in their statements of the intimate character and essential bearings of the observations they have made, or the conclusions they have formed. But, ordinarily, the more profound their investigations of any point, the more careful they are in their references to the opinions of others, lest unhappily they may indelicately misrepresent or mislead multitudes in a phrase, and sow injury broad-cast in an affirmation in which familiarity is assumed with the opinions of the wise men, hard to be obtained and consulted by much the largest number of medical practitioners. The other, for a declaration of entire disrespect for the opinions ot others; aye, more—in defiance of the opinions of a respectable proportion of the profession. Again, for permitting a broad asseveration, involving the opinions of others, to pass without challenge; for not instigating the presentation of proof of the affirmation—thus forcing himself to the wall, or otherwise causing their prosecutor himself to defend the assailed character and aspersed opinions of absent and, under the circumstances, defenseless, members of the profession.

Dr. Dutton knew Dr. Warren's opinions certainly, regarding blood effused in the tissues, as prone to suppuration, because Dr. Warren had so expressed himself. He perhaps had the right to entertain such an opinion, and, entertaining it, had the right, beyond controversy, to declare it; and Dr. Dutton had the right to "stand by" his opinion, "Dr. Warren notwithstanding." Admitting the force of these rights, the question is pertinent, is it ever proper for a disputant to involve others, by a simple affirmation of his own, that they entertain opinion in common with himself on the point in question? or is it ever proper to permit a disputant so referring to the opinion of others to rest easy in the simple declaration, but the rather, is it not incumbent on the other contestant to challenge the assertion, and call for proof?

So much for the ethical point belonging to this discussion, and which is so ordinarily violated as to render most considerations of practical questions nothing more than naked assertions for and against, just as the disputants happen to have expressed opinion.

The record of the session shows that Dr. Bixby took part in canvassing the point, and that he did not fail to specify authority. He did not say that any proportion of professional men sustained one or the other view, but that, having consulted most of the authorities upon the subject, he regarded Nelaton as the highest, "who, at least in the majority of cases, does not believe in the suppurative tendency of retro-uterine hæmatocele;" and he made mention of two instances of blood effused into tissues and in the vicinity of the peritoneum to sustain the principle involved. In both cases the blood remained for a very long while "without undergoing any particular change." They were both instances of hæmatocele of the testicle.

The specific question discussed before the society involves the consideration of changes effected in blood-coagulum, and in the blood discharged from its vessels into shut cavities and into tissues, where it does not come in contact with atmospherical air. Retro-uterine hæmatocele is nothing more nor less than an instance of such hemorrhage.

The specific question discussed also involves some reference to the process of suppuration. "All parts of the body may become the seats of hemorrhage, excepting those which are extremely dense, as the bones, the cartilages, ligaments, tendons, etc. When the hemorrhage is not the consequence of injury or disease, it frequently proceeds from the minute vessels distributed in mucous or serous membranes, or in the parenchyma of organs, as in exhalation or exudation from their extremities or pores. Cases, however, are often met with in which it is very difficult to determine whether the hemorrhage proceeds from exhalation or from rupture of the vessel. Notwithstanding certain resemblances, hemorrhage is far from being the same disease as inflammation. Usually hemorrhage is merely a symptom, contingent upon a variety of affections, the primary ailment being chiefly important. This is the case no less when it takes place as an exhalation from mucous surfaces, as when it occurs from disease of the vessels, or into serous cavities, or the parenchyma of organs.

"The escape of red blood from the vessels takes place in those structures which, owing to their natural laxity, furnish a slight support to the capillaries supplying them. And it must not be overlooked that a change in the state of the tissues will generally, sooner or later, affect the capillaries supplying them, while a lesion of the latter will also affect the state of the former. Discharges of blood seldom take place to any amount excepting in textures which furnish an insufficient support to the capillary vessels, and which imperfectly enable them to withstand the distending power to which they are subjected under various circumstances. All animal tissues are permeable to fluids and gases; hence we are justified in believing that most hemorrhages, not dependent on rupture, are caused by a sort of exosmose, diapedesis, or transudation, by which the elements of the blood escape through the coats of the vessels. All that seems necessary on the part of the vessels, in order to the production of an effusion, is an opening of their pores, so as to admit of a transudation." I have made quotations from Copland and Babington, to place clearly before the mind the distinctiveness between vessels conditioned as in the action called inflammation, and hemorrhage from vessels not supported by the densness, or tenacity, of the tissue they supply, or the capacity of which to retain blood is overcome by its excessive supply, or by agencies that prevent its otherwise escape from the locality at which the hemorrhage occurs. It is true that hemorrhage seldom occurs from vessels involved in inflammatory action, except by the rupture or destruction of the vessels, and ordinarily occurs either by transudation or by excessive enlargement and patuity of the vesssels just at, and approaching, the locality of the hemorrhage; and it is necessary to recognize or admit this difference to apprehend and appreciate the difference in the character of deposit from inflammation and hemorrhage.

While it has been observed that inflammatory action does not necessarily produce pus, it is nevertheless true that inflammatory action is essential to its formation. The condition of the capillaries is peculiar, and the fluid that is discharged is prepared by them with particular properties, in virtue of which it itself undergoes a particular change, and breaks down and assimilates the tissues, with which it is in contact, with itself, and forms pus. And there is the authority of Prescott Hewitt for the affirmation, that "it is by no means a common occurrence to find clots of blood mixed with the effusions resulting from inflammation." Perhaps, in some instances of hemorrhage into shut cavities and into tissues, the accumulated mass may be so excessive as to be a source of irritation, as a foreign body; particularly, and probably only, in subjects who are, for the time being, in relationships peculiarly favoring the development of inflammatory action. At all events, the occurring change is in the tissues surrounding the effused blood and resultant coagulum, and not in the blood, either fluid or coagulated, by which its properties as blood become lost, and the characters of pus assumed. Just here the language of Rindfleisch is pertinent: "When we hear it asserted that pus may be produced in various ways, when we find pus-formation in connective tissue, contrasted with pus-formation on mucous and serous surfaces, or in coagulated blood, etc., we must accept such contrasts with a grain of salt. The great bulk of pus is everywhere formed by the migration of colorless corpuscles from the vessels. As regards the suppuration of an inflammatory exudation, a formative irritation of the stationary corpuscles of connective tissue has been shown to take place, and their breaking up into migratory cells rendered highly probable; while multiplication of the exuded cells by division has been directly observed."

The behavior of blood while yet in contact with living tissue, but relieved from the play of the laws governing it in circulation, can now properly be reviewed. It is well to begin this review of the deportment of the blood, as there is opportunity under such circumstances, in the circulatory system. If it is retarded in its flow, blood deposits fibrine, and forms coagula in its own proper vessels. This occurs in the capillaries, the veins, the arteries and the heart. Retardation, or stoppage of circulation through the capillaries probably is occasioned by emboli from the breakage of a thrombus in larger vessels. However, the injury effected is not in the substance of such transported coagula, but in the changes induced in the surrounding tissues, and "the only clinical phenomena resulting from capillary embolism are such as arise from these tissue changes, and from the blood infection which is apt to accompany them." The point of investigation here has nothing to do with infarctus, "that parenchymatous lesion secondary to capillary embolism," or "the anatomical alteration of a greater or less portion of the parenchyma of a viscus, supervening, through defect of nutrition, on the obturation of the arterial branches supplying it." In what ever manner the blood-clot occurs in capillaries, it has not, within my observation, been charged with deportment differing from the deportment of blood-clot in larger masses in larger vessels. It must be said, though, that specific mention of the deportment of blood-clot in capillaries is hard to find; infarctus for the most part engaging the attention of observers, is made the gravamen of remark by them.

But the obstruction of veins by sanguineous coagula has been extensively observed. In 1839, George Gulliver, F.R.S., said that the labors of continental pathologists, and the reports of dissections by Bright, Arnott, Burrows and Copland, with his own observations, had led him "to believe that the affection is not only more frequent than is generally supposed, but more concerned in disease. The numerous examples of this condition noted in the register of dissections made under the superintendence of Dr. Davy, at Chatham, are sufficient to excite surprise." Gulliver made dissections and performed experiments, from which he felt warranted in saying, that "an extremely low state of the vital powers is favorable to the formation and softening of the coagulum, while clots, in vigorous subjects when the organic force is active, readily become the seat of new formations, acquiring firmness, and remaining for years in the location of their formation; and that extravasated blood is generally soon absorbed. If a vein becomes obstructed with coagulated blood or fibrine, which cannot easily be absorbed or organized in consequence of the very feeble vitality of the patient, the clot may be expected to soften. The softening of coagulated fibrine is an elementary pathological condition of frequent occurrence, distinct from suppuration, and constituting a considerable portion of the cases generally denominated suppurative phlebitis; the softening of clots of fibrine appears, indeed, to be independent of inflammation."

The language of Rindfleish, the pathological histologist, as translated by Baxter, of London, and published by the New Sydenham Society, so recently as 1872, is, "a clot is susceptible of metamorphosis in two different directions—that of organization and that of softening. A more or less vascularization may be demonstrated in every thrombus over eight days old. The vascularization of the clot guarantees it a more durable and organic connection with the body. It becomes henceforth a member of the series of vascular connective tissue. The softening of the thrombi contrasts with their organization in much the same way as suppuration contrasts with organization in the cure of inflammatory products. The resulting liquid contains, as a rule, nothing but granular *debris* and oil globules. True, pus-corpuscles, (even if we include the colorless corpuscles of the blood under this name) occur only in a very small number; and there is no question of a proliferation of corpuscular elements; so that to class the softening of thrombi as suppuration, though it does convey some idea of the naked eye appearances, fails entirely to hit the essential nature of the process." This language of the distinguished pathological histologist, as well as that of Gulliver, is commended to the attention of Dr. Bristow and others, who carelessly—so presumed—employs softening, decomposition and suppuration as interchangeable words, when writing of the deportment of blood-clot.

"Dilatations of veins, notwithstanding their greater frequency, notwithstanding the many anatomical peculiarities which they present, are but a feeble copy of arterial dilatations." In late nomenclature vein dilatations are termed phlebectury; they are more commonly presented for observation under the condition of "varicosity." In this condition the veins are elongated and dilated, necessarily bent, or twisted, and knotted; more or less coagulum forms in their caliber, and the adjacent tissues become involved to a lesser or larger extent. It is far from an ordinary occurrence for the entire caliber of the involved veins to be filled by the coagulum. The older surgeons regarded the process as eminently beneficial when the result is a coagulum so extensive and so firm as to prevent the passage of blood. When the deposition of fibrine, or formation of coagulum, is not enough to retard the flow of blood, it is detrimental, causing an increase of dilatation and extension of coagulum into communicating vessels. But, on the authority of Hodgson, where the coagulum accumulates to such an extent that the canals of the dilated vessels are obliterated, a spontaneous cure is the consequence. Hence the propriety of applying pressure when it can be done with accuracy and power for efficiency without compromising the integrity of other tissues which must receive its force—an objectionable feature of the procedure. Puncturing, ligaturing and cutting the involved vessels each has been proven to be attended with considerable danger, as conducive to inflammatory action in the continuity of the wounded veins; otherwise, it is only when the coagulum acts as a foreign body upon the live tissues that the veins become inflamed, and suppuration results in consequence of the clot. To meet the curative indications, and to avoid objections and difficulties, Richerand made a particular operation, *i.e.*, incision through the adjacent tissues longitudinally with the dilated vessels, for the purpose of securing suppuration, and emptying the vessels of the partially coagulated blood with which they are filled, and thus completely obliterating them, without inflammation spreading along their internal surface, as sometimes occurs under other operative measures. The records show the deportment of blood-clot in veins to be organization and softening. The former conducive to recovery, if it occur before adjacent tissues are involved; the latter, as is true under all circumstances of softening of thrombi, dangerous as a source of blood-contamination, and as furnishing detached particles that become emboli, interfering with the circulation, sometimes to the occasioning of death. Holberton recorded, in 1830, the death of a consumptive patient. *Post mortem* examination revealed, besides various pathological appearances of the veins at different localities, a firm coagulum and false membrane at the mouth of the iliac vein, and an inch and a half down the vessel. And in the twenty-eighth volume of the Medico-Chirurgical Transactions are recorded and noticed numerous cases of obliteration of veins by coagula, in various stages of organization, in some of which the process had been so complete as to have made the involved vessels perfect ligamentous cords—an ultimate that must have required considerable length of time, the circulation having been maintained by compensatory vessels.

When disease in one or more of the coats of an artery, and embracing the whole of its circumference, necessarily admits dilatation or aneurism, the blood is passed on with velocity sufficient, ordinarily, to prevent the formation of coagulum; but in other instances the coats of the vessels are intact, but certainly with weakened integrity, and the blood is coagulated, or partly coagulated, and in layers, and enveloped in a membrane of its own, regarded by some observers as an organizing effort made by the clot itself; denied, however, by others. Benjamin Bell, in describing the progress of aneurism, says: "At last, however, when it becomes large, the pulsation still continues, but the tumor, although soft in some parts, yet in others is firm, and cannot be made to yield much to pressure, part of the contained blood having in this stage of the disease become hard by coagulation." But Copland gives better expression to the facts and explanation: "Coagula do not frequently form in true aneurism as long as the current of blood in the sack continues to be not much obstructed; but when, owing to the narrowness of the mouth, or to retardation of the current of circulation in it, a partial stagnation takes place, coagula then form, frequently in an irregular or confused state, but sometimes in regular layers." There is, though, discrepancy here and in the later teaching of Rindfleisch: "Clots often form in the cavity of the aneurism. The first impulse to their formation is usually given by irregularities of the internal surface. Retardation of the blood current is also an important factor; hence the peculiar frequency with which the blood coagulates in saccular aneurisms with narrow necks. These aneurismal thrombi are always exquisitely laminated; the outer layers, those first deposited, are usually quite decolorized, tough and fibrous, like all clots which have been exposed to continued pressure, no trace of commencing organization can ever be detected. Hence it is that the formation of thrombi in an aneurism, though directly lessening the size of its cavity, seldom leads to a spontaneous cure. This has only been known to occur in aneurisms of small arteries, which have been quite shut off from the parent vessel. For the most part, the filling up of an aneurismal sack with coagula proves inadequate to resist the impulse of the blood.

Aneurismal coagula may also undergo softening, an event which facilitates the detachment of fragments, and increases the risk of embolism." From all of which it is made evident that condensation and coherence, if indeed not organization and softening, is the deportment of blood-clot, when it occurs in arteries; no observer affirming suppuration to occur, except that in adjacent tissues, occasioned by pressure and the force of pulsation.

In the apices of the cardiac ventricles and auricles are a number of pouched recesses, often branched and hidden, from which the blood can never be expelled save by complete contractions. Such recesses do a great deal to help coagulation; it is in them that is found the first beginnings of the process. The contiguous thrombi, filling up and protruding from the inequalities which normally exist in that region, coalesce and produce clots of considerable length and thickness, which take up a great part of the heart's cavities. Their color depends on the time they have existed; and thrombi of any size are seldom met with which are still solid throughout. Such thrombi exhibit an exquisitely laminated composition, with decolorization and softening in the most central parts of the entire clot, from which these changes spread layer by layer to its surface. They may progress until a spherical cyst, filled with fluid, attached to the walls of the cavity by solid rootlets, is the remains of the clot. If the thrombus should by any chance be broken up, the patient will be in imminent peril of embolism, and the accidents which flow from embolism. Such is the teaching of Rindfleisch, and has been introduced here to show that the blood-clot deports itself when formed in the heart, as when formed in other vessels belonging to the circulatory system of the animal economy; in all of these coalescing and cohering, if, indeed, not organizing and softening, but never suppurating.

Sometimes, when a blow is received directly upon the head, and sometimes when the contents of the skull are severely jarred by a fall, received upon the pedal or pelvic extremity of the person, there results effusion of blood within the cranium, which, "if at all considerable, occasions symptoms of pressure immediately, or very soon after the accident; such as stupidity, loss of sense and voluntary motion, laborious and obstructed pulse and respiration. Any inflammatory action, consequent upon such accidents, is marked by symptoms wholly different from those just mentioned, and are such as pain in the head, restlessness, want of sleep, frequent and hard pulse, hot and dry skin, flushed countenance, inflamed eyes, nausea, rigor, and, towards the end, convulsion and delirium. And none of these appear at first, that is, immediately after the accident; seldom until some days are passed." So observed Pott, and subsequent records of observations do not weaken the weight of the more ancient record in this particular. The symptoms of pressure may disappear, and again recur, indicating another hæmorrhage or effusion of blood. Since Virchow, most teachers adopt the opinion of antecedent meningeal inflammation in instances of saculated cerebral hæmatomata; the inflammatory action forming a false membrane, from the vessels of which the hemorrhage occurs, and the vessels of which permeate and organize the clot of the blood discharged from them. This may or may not be so; with all the weight of authority it is as yet but an opinion, and depends for ultimate confirmation, perhaps, upon a more definite conception of inflammation than is at present possessed by medical philosophers. In any event, the teaching does not militate against the purpose of this compilation, the establishing, by reference to recorded observations, that blood exudated, and blood coagulated, organizes, softens, or is absorbed, as seen in hæmatomata within the skull and elsewhere in the living animal economy, and does not suppurate.

Velpeau, claiming precedence in giving close attention to hæmatic tumors, says they are "caused by effusions of blood, and sometimes form for themselves cysts. They are almost constantly surrounded with an irregular cyst when they are situated in the cellular tissue, but sufficiently regular when they are formed on the bursæ mucosæ, or the synovial cavities. And he re-enunciates his opinion, as confirmed by many observations made by himself, that certain polypi of the uterus, tumors of the prostate, and steatomas of the head, breasts, etc., owe their origin to an effusion of blood, or fibrinous concretion." The affirmation of the clinician is that the effusions form for themselves the cysts, and, further, that they form various kinds of tumors; agreeing with the pathological teachings of Rokitansky, who tells us that an exudate susceptible of textural conversion is an organizable exudate; that certain kinds of fibrine, of necessity, become converted into textures, whilst others liquify; that extravasated blood varies in deportment; that blood poured out into cavities and canals, or into textures, is either fluid or in various phases of coagulation; that, of these phases, coagulation, with central or peripheral encysting separation of fibrine is most important as impeding absorption. And enumerates amongst the less immediate effects of hemorrhage, eventual organization of the products effused, callous condensation of the nether layer, and capsular isolation of the hemorrhagic clot. Terminating a paragraph with the positive affirmation that inflammation, resulting in purulent and ichorous products, in parts broken up by hemorrhage, is of rare occurrence, at no time mentioning suppuration as occurring in the extravasated clot.

For a long time the word hæmatocele was employed to designate hemorrhage into, or blood-tumor of, the tunica vaginalis of the testicle; and reference to the older reports and writings of the earlier masters in surgery shows their observations of the deportment of blood, blood-clots, and hemorrhagic deposits in testicular hæmatocele, in accord with the observations of blood, blood-clot, and hemorrhagic deposits occurring at other localities in the animal economy; a permanent or a progressively enlarging tumor, at first soft, afterwards more or less hard, as the degree of coagulation and condensation of the contents may develop the quality; but if the tumor has existed for a long time, the hardness in some measure will be ascribable to the thickening of the tunica. The records of surgery give us cases of such tumors which had progressively enlarged to an enormous size, extending, after many years, even as far as the knee joint, and then not suppurating, but made up wholly of encysted blood debris. Two instances of this character can be readily consulted in the 33d volume of the Transactions of the Medico-Chirurgical Society, 1850, reported by surgeons Bowman and Curling.

Bernutz claims that since a publication of his own, in 1848, the word hæmatocele is applied to other blood-tumors than those of the testicle. Barnes tells us that the term retro-uterine hæmatocele was first proposed by Nelaton, and pelvic hæmatocele by McClintoch. Barnes makes strictures upon the Nelaton designanative, as incomplete, not incorrect as far as it goes; gives preference to the term perimetric hæmatocele, but regards, as does Simpson, that proposed by McClintoch as under all circumstances better than any. There is apology for multiplication of names, as well as for discussion on the force of points involved in determining the condition of a patient under examination for presumed hæmatocele, in the facts that only since 1825, when Velpeau, or 1830, when Recamier gave descriptions of clear cases, that pelvic hæmatocele has been the subject of intimate study, though Bernutz ascribes its first description to Ruysch, as long ago as 1691. The same authority gives to Velpeau the honor of first making a diagnosis, during life, of one of these blood-tumors without having recourse to an exploratory incision, as recently as 1843; whilst Barnes gives the information that seven years subsequently, or in 1850, the brilliant operator and scholastic surgeon, Malgaigne, attempted the enucleation of a supposed fibroid tumor of the uterus, which proved to be a collection of blood; and the operation was followed by a fatal issue; and as late as 1863, Scanzoni published: "We regret not to be in a position, from personal experience, to speak of this disease, for in our certainly extensive and protracted observations we have not been able to diagnose peri-uterine hæmatocele in a single case."

Reference is made by every clinician whom I have been able to consult to the difficulty of determining between a collection of pus and a collection of blood in Douglas' sack. Grailey Hewitt tells us that "the diagnosis of peri-uterine hæmatocele is not easy in all cases. Out of twelve hundred and five cases observed by myself at University Hospital, the affection was diagnosticated in eleven instances." Bernutz affirms that "in the absence of perfectly circumstantial antecedents, more important even for diagnosis than digital examination, there is no one, I believe, who is not, or who may not be at times, deceived;" and quotes a case, reported by a pupil of the operator, as an instance of error made by Nelaton, and selected by Bernutz "from many others of the same nature, in order to show that the surgical examination of the tumor cannot suffice to exclude error, however able they may be to form a diagnosis who content themselves merely with the physical signs presented to them." However interesting and essential the questions of origin of the blood-discharge, and the pathological circumstances with which it may be related, and the symptomatology from the first and successively during the progress of the blood-tumor, I am not now concerned with them. Only the one point of pusification, as belonging to blood deposit, has been attempted by me, it being the issue that was made in the discussion by which the preparation of this paper was suggested. It has been shown, I think, that blood deposit, either in blood vessels, in membranes, or in tissue, will become encysted, will organize, or will be absorbed; that it is not liable to suppurate, or to be connected with suppuration, except sometimes as a mechanical cause of irritation to adjoining tissues, which terminates in suppuration, and sometimes, perhaps, assumes irritative capacity from access of air through exploratory punctures made by examining surgeons. And this is true of pelvic hæmatocele as of blood deposit elsewhere in a living economy, if deference is to be paid to the observations of clinicians on the subject, whose records have been at my command for consultation.

Dutton thought it generally understood that suppuration was the distinctive sign between hæmatocele and pelvic abscess. Niemeyer teaches that "in rare cases the effusion of blood leads to suppuration and abscesses in its vicinity." And again, in regard to puncturing the tumor through the vagina, the same clinician says: "If the entrance of air be not very carefully avoided, this operation may readily induce decomposition of the remaining blood and suppuration in its vicinity." Dutton's expression certainly embraced Barnes' opinion, if the following language be admissible as the criterion by which to reach his opinion on the point; for if he is anywhere more precise it has escaped my attention, unless that be so regarded when he says of the result of puncturing the tumor: "Should pus escape the diagnosis of abscess is tolerably certain." The language about to be introduced, however, conveys to my mind the idea that Barnes thought it generally understood that suppuration was the distinctive sign between hæmatocele and pelvic abscess. Mentioning a case operated upon, and described by a surgeon under the name of "Tumeur sanguine du bassin," he says: "Recamier, believing it to be an abscess, opened it, but instead of pus, dark, half coagulated blood escaped. The patient recovered." And again, Barnes tells us that in two cases regarded by him as pelvic hæmatocele, "the diagnosis was confirmed by the gradual complete disappearance of the tumor without any signs of rupture or of suppuration." It is true that Barnes, I think incautiously, employs the word suppuration as interchangeable, or having the same force, with decomposition, as has already been mentioned here as having been done by others, but who cannot mean suppuration, in a close or proper sense, or they would not have coupled the words, as has been too frequently done by writers in this connection. "But the blood mass may undergo a process of suppurative or decomposing liquifaction," etc. The sometimes decomposition of blood deposit is readily admitted by every one; but true suppuration occurs, the careful Niemeyer teaches, in the "vicinity" of the poured out blood-mass.

Bixby instituted comparison of hæmatocele of the testicle and retro-uterine hæmatocele, in which he is sustained by Bernutz, who says the pathological history of the former exhibits many points of resemblance to that of the analogous affection in the other sex. Bernutz, too, quotes a case determined by Nelaton to be a case of retro-uterine hæmatocele, and punctured by him with the expectation of letting out blood-deposit, but instead he let out a quantity of pus. The discharge of pus is regarded by Bernutz as conclusive proof that Nelaton was in error as to the nature of the patient's condition. The patient made a good recovery. Trousseau records a cure regarded by him as peri-uterine cellulitis, and from the progress of the phlegmonous abscess he deemed it to be expedient, and punctured through the abdomen, expecting to let out pus, "but was greatly surprised to see a quantity of sanguinolent serosity issue from the canula; the remainder of the tumor was composed of fibrous clots. By slow degrees the tumor was absorbed; the patient left the hospital cured, after having exhibited during more than a month signs which led me to form an erroneous diagnosis."

Simpson, in teaching how to distinguish this trouble, says: "An inflammatory tumor, in its earlier stages, is very hard and unyielding, and in its later stages, after suppuration has taken place, it can be felt fluctuating. A blood tumor is comparatively soft and elastic, but the feeling of fluctuation is never very distinct in the mass of the swelling." Simpson, though, is very particular to charge his pupils, "Always, however, bear in remembrance, further, that you may have both diseases present, and that large pelvic hæmatoceles often excite inflammation, and thus become combined with pelvic cellulitis and suppuration." A different form of expressing the same idea as that of Niemeyer—suppuration in the "vicinity" of the blood-deposit.

Grailey Hewitt, writing of the results of pelvic hæmatocele, says: "Absorption of the coagulum is the most common event, and this is the most favorable termination. In some cases the blood-tumor bursts into adjacent viscera. The bowel is the outlet most commonly chosen, and the syrupy contents of the cavity then escape by stool, or flesh-like masses are passed in this manner from time to time, the tumor diminishing in size as this goes on. The tumor may burst into the vagina; it may burst also into the peritoneum. This latter termination is the most unfavorable," etc. Very clearly Hewitt entertained no idea of puscification as belonging to, though fully appreciating it as a complication of, a pelvic hæmatocele.

Thomas, in a paragraph containing his views of the terminations of pelvic hæmatocele, says: "The tumor generally disappears by absorption, is discharged by the rectum or vagina, or remains a hard, indurated mass for years afterwards. Discharge is most frequently followed by recovery, but sometimes putrefaction occurs in the walls of the sack, septicæmia takes place, and death ensues."

